# Improvement of a Real-Time Reverse Transcription–Polymerase Chain Reaction Assay for the Sensitive Detection of the F Gene of Avian Orthoavulavirus-1 (AOAV-1)

**DOI:** 10.3390/vetsci10030223

**Published:** 2023-03-14

**Authors:** Prerana Bhande, Brigitte Sigrist, Linard Balke, Sarah Albini, Nina Wolfrum

**Affiliations:** 1 Section of Poultry and Rabbit Diseases, Institute for Food Safety and Hygiene, Vetsuisse Faculty, University of Zurich, 8057 Zurich, Switzerland; 2 Bachelor’s Program, University College Utrecht, Utrecht University, 3584 Utrecht, The Netherlands

**Keywords:** avian orthoavulavirus-1 (AOAV-1), pigeon paramyxovirus-1 (PPMV-1), Newcastle disease, diagnostics, real-time reverse transcription–polymerase chain reaction assay (rRT-PCR)

## Abstract

**Simple Summary:**

Avian orthoavulavirus-1 (AOAV-1) can cause Newcastle disease in poultry, and thus lead to massive economic losses worldwide. Compared to the original assay, the adapted real-time reverse-transcription PCR presented here proved to be more sensitive for detecting currently circulating AOAV-1 strains, in particular the pigeon-type strains (pigeon paramyxovirus-1). This highlights the importance of constant monitoring of existing methods.

**Abstract:**

Avian orthoavulavirus-1 (AOAV-1) is the causative agent of Newcastle disease in poultry. This highly infectious disease causes large economic losses annually and worldwide. AOAV-1 does not only infect poultry, but it has a very broad host range and has been detected in over 230 bird species to date. A distinct group of viral strains within AOAV-1 are pigeon-adapted strains, also named pigeon paramyxovirus-1 (PPMV-1). AOAV-1 is transmitted through the feces of infected birds and secretions from the nasal and oral cavities and eyes. It is worth mentioning that wild birds can transmit the virus to captive birds, especially feral pigeons to poultry. Therefore, early and sensitive detection of this virus—including the monitoring of pigeons—is of utmost importance. A variety of molecular methods for the detection of AOAV-1 already exist, but the detection of the F gene cleavage site of currently circulating PPMV-1 strains has not proven to be particularly sensitive or suitable. As presented here, by modifying the primers and probe of an already established real-time reverse-transcription PCR, the sensitivity could be increased, allowing for a more reliable detection of the AOAV-1 F gene cleavage site. Furthermore, it becomes clear how important it is to constantly monitor and, if necessary, adapt existing diagnostic procedures.

## 1. Introduction

The avian orthoavulavirus-1 (AOAV-1) is an RNA virus of the family *Paramyxoviridae*, genus *Orthoavulavirus* [1]. Certain virulent strains of AOAV-1, formerly known as avian paramyxovirus-1 (APMV-1), can cause Newcastle disease (ND) in poultry; one of the most important infectious diseases in chicken worldwide. Clinical signs in infected chickens include sudden death, along with neurological, respiratory, and gastrointestinal signs. In addition, a decrease in feed intake is often observed, as well as a decrease in laying performance, and poor eggshell quality (thin-shelled, discolored or shell-less eggs) [2]. Other AOAV-1 strains are predominantly found in pigeons where they can cause clinical disease with symptoms similar to ND in poultry and mass mortality. The latter group of viruses belong to the AOAV-1 pigeon type, also called pigeon paramyxoviruses-1 (PPMV-1). PPMV-1 can again be transmitted to chickens, where it can cause ND [3,4]. Thus, it is extremely important to be able to reliably detect virulent PPMV-1 strains as well. If there is a certain amino acid sequence at the cleavage site of the fusion protein, this is an indicator of virulence. Characteristic of this sequence are several basic amino acids at positions 113–116 and a phenylalanine at position 117 [5]. AOAV-1 can be diagnosed by the detection of viral nucleic acids by real-time reverse-transcription PCR (rRT-PCR). There are several established methods that detect the M, L, or NP genes, but this does not allow conclusions to be drawn about virulence or pathogenicity [6,7,8]. However, a detection of the F gene cleavage site allows first suspicion of a virulent form, which finally needs to be confirmed by an analysis of the amino acid sequence. Almost two decades ago, Wise and colleagues published an rRT-PCR for the detection of AOAV-1 RNA including a primer/probe set particularly targeting the F gene cleavage site enabling the detection of potentially virulent AOAV-1 isolates [6]. Routine diagnostic as well as proficiency testing schemes revealed that the detection of the F gene cleavage site using this primer/probe set was not very sensitive and thus not reliable in detecting currently circulating strains. Continuous evaluation and, if necessary, adaptation of existing detection methods is particularly important because AOAV-1 is constantly changing. Presumably, the currently circulating AOAV-1 evolved from viruses with low virulence by mutation of the fusion protein cleavage site, and cross-species adaptations continue to change the viruses [9,10].

Here, we present a modified rRT-PCR that proved to be more sensitive than the underlying Wise-based F gene assay and much more reliable in detecting the herein tested AOAV-1 strains.

## 2. Materials and Methods

### 2.1. Virus Strains and Field Samples

RNA extracted from three reference strains (PPMV-1: 19VIR1689-2, APMV-1_2: 00VIR2875, APMV-1_3: 00VIR3282; Istituto Zooprofilattico Sperimentale delle Venezie, IZSVe, Legnaro, Italy) was used for cloning of positive controls (Table 1). The vaccine strain NDV VG/GA (IZSVe, Legnaro, Italy) was used for cloning a negative control. A total of 40 clinical samples (extracted RNA from kidneys from feral pigeons) were used for validation of the assay and compared with the Wise-based F gene primer/probe set. The samples were known to be AOAV-1 positive due to previous analysis by rRT-PCR targeting the M-gene [6]. Furthermore, the HN- and F genes of four of these samples (#0147, #0228, #0297, #0382) have been sequenced. This revealed the genotype VI.2.1.1.2.2 for all of them and the amino acid sequence of the F gene cleavage site RRQKR*F which is characteristic for a virulent AOAV-1 [3]. In another four samples (#261, #306, #345, #359), only the cleavage site of the F gene was sequenced, and also resulted in RRQKR*F for all of them.

### 2.2. Primers and Probe

**Figure 1:**
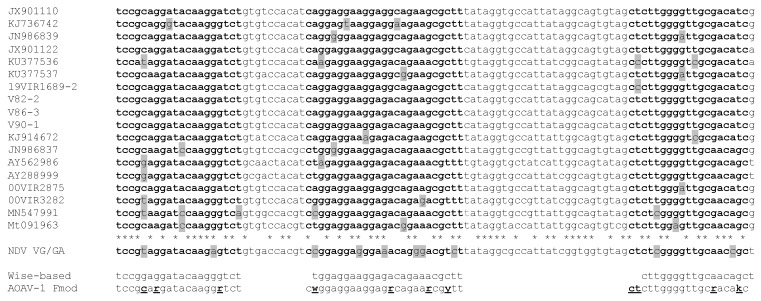
Primer and probe design. Alignment of the partial AOAV-1 F gene sequences (positions 296 to 396). Boldface: sequences corresponding to the primer and probe binding regions; grey shaded: nucleotides mismatching with the modified (AOAV-1 Fmod) primer/probe sequence; boldface underlined: nucleotides replaced compared to the Wise-based sequences; *: conserved nucleotides.

F gene sequences of 18 mainly European AOAV-1 isolates from pigeons (n = 11) and chickens (n = 7) and one vaccine strain (VG/GA) F gene sequence were aligned using Clustal Omega (Table 1, Figure 1). The original primers and probe sequences published by Wise et al. were modified to better cover the aligned sequences [6]. To achieve this, mixed nucleotides were inserted at some positions (Figure 1, Table 2).

### 2.3. Generation of Recombinant Plasmids and Synthesis of Reference RNA Transcripts

According to the manufacturer’s protocol, the viral RNA of the three reference strains and one vaccine strain (VG/GA; Istituto Zooprofilattico Sperimentale delle Venezie (IZSVe)) was transcribed into cDNA by the Reverse Transcription System (Promega, Madison, WI, USA) using a mixture of random hexamer and oligo(dT)15 primers. Using primers F+ 5′-ATCATAGTCAAGTTGCTCCC-3′ and F- 5′-GCAGCYGCTGTTATCTG-3′, a 236 bp fragment of the F gene, spanning the target sequence, was amplified. This fragment was cloned into the pCR2.1-TOPO vector (Invitrogen, Thermo Fisher Scientific, Waltham, MA, USA). By sequencing (Microsynth, Balgach, Switzerland), the successful cloning was verified. Through a BamHI digestion (NEB, Ipswich, MA, USA), the vector constructs were linearized. Except for an overnight incubation at 37 °C, in vitro transcription from the T7 promoter was performed using the MEGAscript T7 kit (Ambion, Thermo Fisher Scientific, Waltham, MA, USA) according to the manufacturer’s instructions. To remove residual DNA, the in vitro transcribed RNA was first treated with a nickase (Nb.BssSI, NEB, Ipswich, MA, USA) and then with Turbo DNase (Ambion, Thermo Fisher Scientific, Waltham, MA, USA). Subsequently, LiCl precipitation was performed, and the RNA concentration was determined spectrophotometrically using the NanoDropTM 2000 spectrophotometer (Thermo Fisher Scientific, Waltham, MA, USA). The following formula was used to calculate copy numbers: Copy number = (mass RNA [g]/molar weight [g/mol]) × Avogadro’s number. A real-time PCR (without the RT step) was performed with tenfold serial dilutions of RNA to check for the absence of DNA in the in vitro transcribed RNA (data not shown).

### 2.4. Real Time RT-PCR Assays

Optimal annealing temperatures and primer/probe concentrations for the modified F gene assay (AOAV-1 Fmod) were determined empirically. The 25 µL PCR reaction contained 1x reaction mix, 0.25 µL RT mix (both QuantiTect Probe RT-PCR Master Mix, Qiagen, Hilden, Germany), 0.3 µM of each primer, 0.25 µM probe, and 5 µL in vitro-transcribed reference RNA or 5 µL RNA extracted from clinical samples in DEPC-treated H_2_O. Reverse transcription and amplification were performed on a QuantStudio 5 Real-Time PCR System (Applied Biosystems, Thermo Fisher Scientific, Waltham, MA, USA) with the following conditions: 1 × 50 °C for 30 min, 1 × 95 °C for 10 min, 45 cycles of 95 °C for 15 s, 53 °C for 1 min, 70 °C for 1 min. The Wise-based assay was tested using the originally published conditions (annealing temperature 58 °C, 0.4 µM reverse primer, 1.2 µM forward primer, 0.24 µM probe) and using the optimized conditions for the AOAV-1 Fmod assay [6]. This comparison revealed a better performance using the optimized conditions for the AOAV-1 Fmod assay (annealing temperature 53 °C, 0.3 µM of each primer, 0.25 µM probe), which resulted in even higher sensitivity than described by Wise et al. (10^3^ vs. 10^4^ copies). The final comparison of the AOVA-1 Fmod assay with the original Wise-based F gene assay was ultimately performed under uniform conditions (as described above). The target was in vitro-transcribed reference RNAs (PPMV-1, APMV-1_2, APMV 1_3) corresponding to 10^2^ to 10^7^ copies per reaction. The target sequence for both assays spans 100 nucleotides.

### 2.5. Determination of Analytical Sensitivity

One-step rRT-PCRs were performed with defined copy numbers of the in vitro-transcribed reference RNAs (tenfold serial dilutions corresponding to 10^2^ to 10^7^ copies per reaction) to determine the analytical sensitivity of the AOAV-1 Fmod and the original Wise-based F gene assay. Only samples that could be repeatedly detected with a C_T_ value below 41 were considered positive. Three independent experiments were performed in duplicate. Using Excel, standard curves of the mean C_T_ values of all three experiments were generated and a regression analysis was performed.

## 3. Results

### 3.1. Primer and Probe Modification

In each primer and the probe, three to four nucleotides were modified (Table 2). To confirm the specificity of the primers and probe, the Basic Local Alignment Search Tool (BLAST) of the National Center for Biotechnology Information (NCBI) was used with default settings. No off-targets for the forward primer and the probe were revealed. Merely, the reverse primer showed 98.5% nucleotide identity with a sequence stretch of chromosome 16 of the wood mouse (*Apodemus sylvaticus*).

### 3.2. Analytical Sensitivity of the AOAV-1 Fmod rRT-PCR Assay

**Figure 2:**
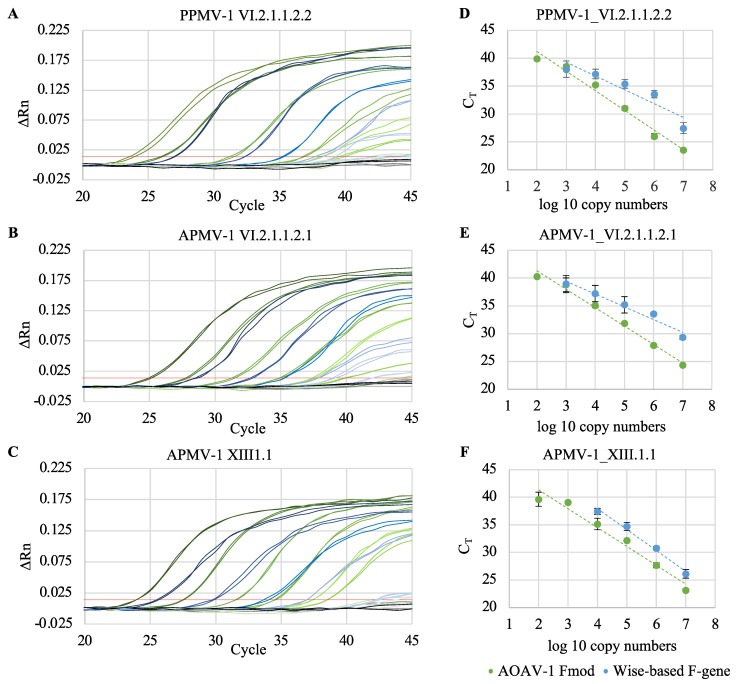
Comparison of the new modified rRT-PCR assay (AOAV-1 Fmod) with the original Wise-based rRT-PCR assay. Serial tenfold dilutions of quantitated in vitro-transcribed RNAs (10^2^ to 10^7^ RNA copies) of different AOAV-1 reference strains were used as templates. (**A**–**C**) Amplification plots. Red line: threshold (0.014), gray/black line: negative control, green lines: AOAV-1 Fmod assay, blue lines: Wise-based assay. For each reference RNA one representative experiment (in duplicates) out of three independent experiments is shown. (**D**–**F**) Regression analysis. Results are presented as mean CT values of three independent experiments, each performed in duplicates.

The analytical sensitivity of the AOAV-1 Fmod and the Wise-based F gene assay was evaluated by performing the rRT-PCRs with serial tenfold dilutions of quantitated in vitro-transcribed reference RNAs. Using the AOAV-1 Fmod assay, 10^2^ copies of PPMV-1, APMV-1_2, APMV-1_3 RNA were detected, respectively. The original Wise-based F gene assay detected 10^3^ copies of PPMV-1, APMV-1_2, and 10^4^ copies of APMV-1_3 RNA (Figure 2, Table 3). The rate of amplicon generation (efficiency, E), for the AOAV-1 Fmod assay ranged from 92.2% to 100.2%, and the linearity of the standard curve (coefficient of determination, r^2^) ranged from 0.98 to 0.99 (Table 3). Values reached with the Wise-based F gene assay were E between 83.7% and 172.7% and r^2^ between 0.87 and 0.99. None of the assays (AOAV-1 or Wise-based F gene) reliably detected the F gene target sequence of the vaccine strain VG/GA (data not shown).

### 3.3. Detection of the F Gene of AOAV-1 in Field Samples

The AOAV-1 Fmod assay was used for the detection of the AOAV-1 F gene in 40 clinical samples with known AOAV-1 status. They all tested positive for the AOAV-1 M-gene [3]. In addition, they had previously been examined with the Wise-based F gene assay. Consistently, all samples were detected compared to the Wise-based F gene assay, which only detected 12 out of 40 samples as F gene-positive (Table 4).

## 4. Discussion

The aim of this project was to improve an already existing rRT-PCR enabling a more sensitive and reliable detection of currently circulating AOAV-1 strains. The first goal was the modification of the primers and the probe. In silico sequence analysis revealed one potential off-target effect for the reverse primer. It showed 89.5% nucleotide identity with a sequence stretch of chromosome 16 of the wood mouse (*Apodemus sylvaticus*). Since it is unlikely that samples submitted for AOAV-1 testing are contaminated with DNA (or RNA) of the wood mouse, this off-target effect is negligible.

When comparing the modified AOAV-1 Fmod assay with the previously published Wise-based F gene assay, the performance of the AOAV-1 Fmod assay was better. All tested references were detected with both assays (genotypes VI.2.1.1.2.2, VI.2.1.1.2.1, XIII.1.1). In silico sequence analysis, however, suggests that the primers and probe also allow detection of genotype XXI.1.1 using the AOAV-1 Fmod assay. In contrast, using the Wise-based F gene primer/probe set, the detection of this genotype is unlikely, since the target sequence contains one mismatch to the forward primer and three mismatches to the probe and the reverse primer each. In recent decades, genotype XXI.1.1 has been increasingly detected in addition to genotype VI.2.1.2, which is predominant in Europe [13]. Using the modified AOAV-1 Fmod assay, the limit of detection was decreased by one to two log units and the efficiency and the linearity (E: 92.2–100.2%, r^2^: 0.98–0.99) were both in the desired range for rRT-PCR assays (E: 90–100%, r^2^: >0.98) [14,15], in contrast to the Wise-based F gene assay (E: 83.7–172.7%, r^2^: 0.87 and 0.99).

Using the modified AOAV-1 Fmod assay, the F gene cleavage site was detected in all 40 tested clinical samples derived from pigeons. The C_T_ values were consistently lower than those for the M-gene rRT-PCR, suggesting even higher sensitivity under the given conditions. In contrast, using the Wise-based F gene assay, only 12 out of 40 samples were tested positive for the F gene cleavage site. The possibility that false-positive samples were detected by the modified assay cannot be completely excluded but is unlikely. All samples previously tested positive by rRT-PCR targeting the M-gene [3]. In four samples, where the F gene was detected only by the modified assay but not by the Wise-based assay, the F gene cleavage site was sequenced and revealed a multibasic amino acid motif characteristic of virulent AOAV-1. Furthermore, the negative control (NDV vaccine strain) was not detected by the modified assay, also supporting specificity.

Transmission of AOAV-1 from wild birds, particularly from pigeons to chickens, has been shown to be possible [3,4]. As free-range poultry production is increasing in many regions of the world, the risk of pathogen transmission from wild birds to domestic poultry is also increasing. Thus, disease monitoring of wild birds is becoming even more important. The rRT-PCR presented here proved to be sufficiently sensitive to detect potentially virulent forms of AOAV-1 and is therefore a valuable tool to systematically screen pigeons for PPMV-1.

## 5. Conclusions

The modified AOAV-1 Fmod assay presented herein was shown to be more sensitive than the original Wise-based F gene assay. Furthermore, this work highlights the need for constant monitoring (e.g., by proficiency testing schemes), and adaption or modification of established methods to ensure the most sensitive detection of particular genes of important pathogens.

## Figures and Tables

**Table 1:** Avian orthoavulavirus-1 isolates used for F gene sequence alignment.

No.	Host	Country	Year	Genotype ^a^	Gen Bank No. or Internal Identifier
1	Pigeon *(Columba livia domestica)*	Belgium	1998	XXI.2	JX901110.1
2	Pigeon *(Columba livia domestica)*	Germany	1998		KJ736742.1
3	Pigeon *(Columba livia domestica)*	Ireland	2004	XXI.2	JN986839.1
4	Pigeon *(Columba livia domestica)*	Belgium	2011		JX901122.1
5	European turtle dove *(Streptopelia turtur)*	Italy	2012		KU377536.1
6	Pigeon *(Columba livia domestica)*	Italy	2014		KU377537.1
**7**	**Pigeon *(Columba livia domestica)***	**Italy**	**2019**	**VI.2.1.1.2.2**	**19VIR1689-2, PPMV-1**
8	Pigeon *(Columba livia domestica)*	Switzerland	2021	VI.2.1.1.2.2	V82-2
9	Pigeon *(Columba livia domestica)*	Switzerland	2021	VI.2.1.1.2.2	V86-3
10	Pigeon *(Columba livia domestica)*	Switzerland	2021	VI.2.1.1.2.2	V90-1
11	Pigeon *(Columba livia domestica)*	Ukraine	2011	XXI.1.1	KJ914672 [11]
12	Chicken *(Gallus gallus)*	Netherlands	1993	VII.2	JN986837.1
13	Chicken *(Gallus gallus)*	USA	1993	XIX	AY562986.1
14	Chicken *(Gallus gallus)*	Mexico	1996	V.1	AY288999.1
**15**	**Chicken *(Gallus gallus)***	**Italy**	**2000**	**VI.2.1.1.2.1**	**00VIR2875, APMV-1_2**
**16**	**Chicken *(Gallus gallus)***	**Italy**	**2000**	**XIII.1.1**	**00VIR3282, APMV-1_3**
17	Chicken *(Gallus gallus)*	Belgium	2018		MN547991.1
18	Chicken *(Gallus gallus)*	Botswana	2019		MT091063.1
**19**	**NDV VG/GA vaccine strain**			**I.1.1**	

^a^ according to Dimitrov [12]. Bold: Reference strains that were used as positive and negative controls.

**Table 2:** Primers and probes. Original Wise-based F gene and modified primers and probe for AOAV-1 F gene detection by rRT-PCR.

	Name	Wise-Based F Gene 5′ → 3′	Name	Modified (AOAV-1 Fmod) 5′ → 3′
**For**	F+4839	TCCGGAGGATACAAGGGTCT	F+4839_m2	TCCG**C**A**R**GATACAAGG**R**TCT
**Probe**	F+4894	AAGCGTTTCTGTCTCCTTCCTCCA	NDF_FAM_m4	AA**R**CG**Y**TTCTG**Y**CTCCTTCCTCC**H**G
**Rev**	F−4939	AGCTGTTGCAACCCCAAG	F−4939_m3	G**M**TGT**Y**GCAACCCCAAG**AG**

Bold: modified nucleotides.

**Table 3:** Regression analysis of standard curves and sensitivity of the AOAV-1 F gene and the Wise-based F gene assay. r^2^: coefficient of determination; E: efficiency.

		Target		
Parameter	Primer/Probe Set Genotype	PPMV-1 VI.2.1.1.2.2	APMV-1_2 VI.2.1.1.2.1	APMV-1_3 XIII.1.1
r^2^	Modified (AOAV-1 Fmod)	0.98	0.99	0.97
	Wise-based F gene	0.87	0.96	0.99
E	Modified (AOAV-1 Fmod)	92.2%	100.2%	96.3%
	Wise-based F gene	153.3%	172.7%	83.7%
Detection limit ^a^	Modified (AOAV-1 Fmod)	10^2^	10^2^	10^2^
	Wise-based F gene	10^3^	10^3^	10^4^

^a^ lowest copy number that could be reproducibly detected.

**Table 4:** Clinical samples of feral pigeons. RNA extracted from kidney previously tested positive for the AOAV-1 M-gene [3] was tested with the AOAV-1 F gene and the Wise-based F gene assay. **C_T_**: threshold cycle value, nd: not detected.

No.	Pigeon No.	C_T_ AOAV-1 Fmod	C_T_ Wise-Based F Gene	C_T_ M-Gene	No.	Pigeon No.	C_T_ AOAV-1 Fmod	C_T_ Wise-Based F Gene	C_T_ M-Gene
1	0115	32.15	nd	32.50	21	0296	27.16	33.71	28.96
2	0116	34.15	nd	34.01	**22**	**0297 ^a^**	**18.45**	**27.89**	**19.32**
**3**	**0147 ^a^**	**17.75**	**24.96**	**19.65**	23	0305	23.82	29.84	25.77
4	0148	26.25	32.01	28.35	24	0306 ^a^	30.15	nd	33.14
5	0149	20.06	26.63	22.06	25	0311	21.50	26.70	22.85
6	0172	34.85	nd	35.20	26	0315	25.64	nd	26.82
7	0212	35.79	nd	38.41	27	0316	32.98	nd	34.08
8	0217	35.51	nd	38.83	28	0345 ^a^	27.69	nd	29.66
9	0218	18.48	25.89	20.45	29	0352	33.00	nd	36.54
10	0227	21.80	28.32	26.06	30	0355	30.80	nd	33.24
**11**	**0228 ^a^**	**20.05**	**26.19**	**20.85**	31	0359 ^a^	32.86	nd	34.11
12	0230	23.57	28.83	24.22	32	0360	35.28	nd	39.63
13	0234	34.22	nd	36.13	33	0366	36.93	nd	40.34
14	0236	35.99	nd	38.58	34	0368	33.87	nd	39.16
15	0259	34.48	nd	39.79	35	0369	34.88	nd	35.79
16	0261 ^a^	29.31	nd	30.58	36	0371	38.02	nd	41.50
17	0263	27.57	32.73	28.78	37	0374	33.85	nd	35.17
18	0286	33.76	nd	35.47	38	0377	31.96	nd	34.04
19	0289	34.17	nd	36.41	39	0379	32.19	nd	33.49
20	0293	33.62	nd	34.78	**40**	**0382 ^a^**	**19.20**	**nd**	**19.42**

^a^ F gene cleavage site RRQKR*F. Bold: genotyped as VI.2.1.1.2.2 by sequencing of the F- and HN-genes.

## Data Availability

Not applicable.
